# Genomics and epilepsy: Opportunities to improve understanding and management

**DOI:** 10.1111/dmcn.16472

**Published:** 2025-08-20

**Authors:** Sanjay M. Sisodiya

**Affiliations:** ^1^ Department of Clinical and Experimental Epilepsy UCL Institute of Neurology London UK; ^2^ Chalfont Centre for Epilepsy Chalfont St Peter Buckinghamshire UK

## Abstract

Genomics has advanced our understanding of epilepsy through the discovery of the causes of many hundreds of different individual syndromes and the discovery of common variants contributing to the epilepsy risk. Many genomic research studies and clinical genetic laboratories now use advanced sequencing methods, including whole‐genome sequencing studies. Such work generates significant amounts of data beyond a possible causal variant alone and can contain information about more complicated genomic contributions to the phenotype, including oligogenic and polygenic influences, modifiers, risk factors for traits such as postictal psychosis, and pharmacogenomic variants of importance to adverse reactions. Extraction of such data will help improve the characterization of each individual's epilepsy, potentially from diagnosis onwards. Newer methods, such as single‐cell studies and combinations of genomic data with other data types are now being used. Genomics may also help protect people with epilepsy from climate change challenges. Opportunities from genomics will continue to enhance our understanding and management of epilepsy.

## Graphical abstract

Accessing the entire genome, beyond any single causal variant for an individual's condition, may reveal additional genetic information of value in the understanding or care for that person. Combining such data with additional sources of information from that individual may enhance understanding and care further.

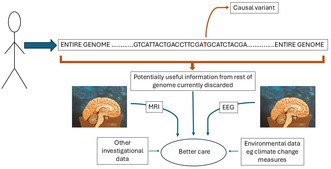

Plain language summary: https://onlinelibrary.wiley.com/doi/10.1111/dmcn.70015

**Abbreviations**
4‐AP: 4‐aminopyridine
DEE: developmental and epileptic encephalopathy
PRS: polygenic risk score
WGS: whole‐genome sequencing

**What this paper adds**

- Sequencing data contain useful information beyond causal variants alone.
- Epilepsy syndromes considered fully explained by single variants may have other genetic contributions.
- Combining genomic and other data may yield additional clinically useful insights.

How much information does the human genome contain? How much of this information is useful in the health care setting? With the advent and growing implementation of whole‐genome sequencing (WGS) in clinical practice and research, these are appropriate questions to ask. Empirical exemplar answers to these questions are considered through the lens of the epilepsy syndromes—important, common, and often burdensome conditions. In a paediatric setting, these two questions may be seen to be particularly important. Answers may assist in counselling families about what the future might hold for their children with epilepsy. Answers to those questions are just as important for adults with epilepsy, regardless of whether their epilepsy had or did not have childhood onset. Lessons emerging may be of broader relevance still to all who seek a better understanding of the condition affecting the person in front of them in the clinic.

## WHAT HAVE WE ACHIEVED WITH GENOMICS SO FAR, AND WHAT REMAINS TO BE DONE?

### The power of genomics

Undoubtedly, genomics has already enabled enormous progress in our understanding of epilepsy. Genomic sequencing can explain why a group of carefully characterized patients with a shared core phenotype are so similar, identifying the gene that may carry pathogenic variants to be found in each such patient. New conditions might be identified in this way. From this starting point, rational exploration of disease biology, and potential new, specific, or ‘precision’ treatments may emerge, although currently fewer than we might hope. Reverse phenotyping may reveal new conditions associated with variants in a given gene identified through statistical or other reasoned approaches.^1^ A large and growing number of genes carrying various types of rare pathogenic variants have thus been associated with epilepsy,^2^ especially with developmental and epileptic encephalopathies (DEEs). Useful and regularly updated websites list these.^3^ For the more common types of epilepsy, successively bigger genome‐wide association studies run by the International League Against Epilepsy Consortium on Complex Epilepsies have identified many associated common variants, especially for generalized epilepsy.^4^ Even larger studies are in progress. Overall, such work has enabled better understanding of both common and rare types of epilepsy. Moreover, this information has facilitated new types of studies. For example, the calculation of polygenic risk scores (PRS) based on genome‐wide association studies shows that common genomic variation may contribute to penetrance and phenotypic diversity in families,^5^, ^6^ and to brain structural variation measured using neuroimaging.^7^ The application of next‐generation sequencing, increasingly through WGS, early after diagnosis will help prevent long diagnostic and therapeutic odysseys^8^ and should result in better clinical outcomes.

### An example: *KCNA2* gain‐of‐function developmental and epileptic encephalopathy

One example illustrates the power and limitations of the current use of genomics. After the discovery of rare variants in *KCNA2* as the cause of several types of epilepsy,^9^ 4‐aminopyridine (4‐AP) emerged as an informed treatment choice with potentially specific value in *KCNA2* gain‐of‐function‐related DEE,^10^ showing reversal of gain of function in vitro. A young man with *KCNA2* DEE because of the R297Q gain‐of‐function variant was treated with 4‐AP aiming to halt deteriorating gait and cognitive function. He was already seizure‐free on valproate and had experienced valproate‐related adverse effects. Within a few weeks of treatment at 20mg/day, there was improvement in gait and simple measures of cognitive performance, in keeping with findings from the larger published series.^10^ The patient, who is able to express this view, and his family, were pleased with the outcome. This scenario might be considered validation of the model of gene discovery, revelation of aspects of pathophysiology, and a rational treatment approach, with ensuing desired outcomes—a success for genomics. However, in an older patient with the same, but more severe, phenotype because of an adjacent mutated residue (L298F) also causing gain of function, two separate trials of 4‐AP at just 10mg/day (with different combinations of co‐prescribed antiseizure medications during the two trials) both resulted in a doubling of the frequency of generalized tonic‐clonic seizures necessitating cessation of 4‐AP. However, a child with the same variant (L298F), benefited from 4‐AP.^10^ It is difficult to call 4‐AP a precision treatment in this context because we do not actually know how 4‐AP is acting in vivo in the whole‐brain system. Genomics alone has not yet provided a precise explanation for the contrasting effects of 4‐AP in these patients. These outcomes are not atypical of precision treatments in many DEEs, with initial excitement not supported by subsequent trials. We should undoubtedly embrace rational new treatments, but we need to proceed with caution.

### DEE: More than monogenic?

Extending this concept, there are many aspects of most DEEs that are unexplained by the discovery of the ‘causal’ monogenic variant found in many DEEs. The most clearly understood DEE, Dravet syndrome, has the same clinically recognizable core phenotype in almost every affected patient, most of whom carry loss‐of‐function variants in *SCN1A*. But those variants, even allowing for their different classes (e.g. loss‐of‐function vs missense, with a consequence that is rarely empirically determined), do not adequately explain the broad diversity of phenotypes seen in Dravet syndrome, or indeed in a wide range of other ‘monogenic’ DEEs.^11^ Our understanding of drug response, treatment adverse effects, multimorbidities, environmental influences, and outcomes, even in the ‘explained’ DEEs, is also limited. Genomics has not yet explained everything,^11^ assuming it will ever be able to. There is much left to do. Current short‐read WGS identifies a genetic cause in up to 50% to 60% of selected cases. Other methods, including long‐read sequencing and optical genome mapping, are emerging and may increase the proportion of cases successfully genetically solved. But even before such advances, we have largely been contented with finding a genetic cause for an epilepsy syndrome, and then we have usually ignored the rest of the genome. Perhaps we can make better use of that additional data, working the genome harder. Some examples are considered in the next sections.

## WORKING THE GENOME HARDER: WHAT ELSE CAN WE GLEAN FROM IT?

### Oligogenic, polygenic, and non‐coding influences and causes

Many rare types of epilepsy are considered to be ‘monogenic’, in that the phenotype is considered fully or very largely attributable to the underlying pathogenic or likely pathogenic variant (epilepsy types related to structural variants are not included here). The phenotypic spectrum related to such monogenic causes is often surprisingly wide. For example, after initial publication of the discovery of a gene linked to a DEE, additional cases are published through which the phenotype is typically broadened. This often results in notable overlap across DEEs and with other conditions. Dravet syndrome is a good example. Beyond a ‘core’ phenotype, sufficiently distinctive as to lead to a clinical definition, there is a wide spread of severity in many aspects of the condition, such as clinical characteristics (cognitive, speech and language, swallowing function), brain structural imaging findings, response to treatments, and longer‐term outcomes, among others. The question must be asked as to whether this is sensible, or whether other explanations should be sought for these apparently extended phenotypes. Should the boundaries of a particular genetic epilepsy be more sharply delineated? In some circumstances, the diverse outcomes of different variants in the same linked gene may be relevant, allelic disorders related to *ATP1A3* being a good example. But variant identity, genic location, or variant class may not explain phenotypic diversity, with *SCN1A* variants in Dravet syndrome being an example. Other explanations for phenotypic breadth might include genetic (oligogenic, polygenic, other genetic) influences acting with a pathogenic variant of major effect.

Oligogenic contributions to DEEs have been determined from a statistical genetics perspective.^12^, ^13^ Individual examples of composite phenotypes related to oligogenic causation have been published, for example, the variation in *DEPDC5* seen in a patient with Dravet syndrome because of a pathogenic variant in *SCN1A*, who also has epileptogenic focal cortical dysplasia,^14^ or a pathogenic variant in *ALPL* in a patient with Unverricht–Lundborg disease caused by biallelic variants in *CSTB*.^15^ Other examples will probably emerge from WGS studies, if we make sure to look for them.

Polygenic contributions have also been shown in presumed monogenic types of epilepsy.^16^ In Dravet syndrome specifically, PRS for intelligence, longevity, and epilepsy were significantly different to those seen in non‐*SCN1A*‐related epilepsy without other known monogenic causes;^14^ a polygenic background influences epilepsy phenotype in monogenic families.^5^, ^6^ An elevated PRS for schizophrenia was shown in people with epilepsy who developed postictal^17^ or drug‐related psychosis.^18^ Larger studies are needed to determine the clinical utility of such PRS findings, but clinical uses could include more informed selection of antiseizure medications, better risk profiling for adverse outcomes like sudden unexpected death in epilepsy, and better counselling for possible outcomes from specific gene‐based therapies. Other such examples are likely to emerge, provoking reconsideration of what we mean by monogenic.

Non‐coding variation has received comparatively little attention in epilepsy. Pathogenic variation in the non‐coding spliceosomal small nuclear RNA gene *RNU4‐2* is a frequent cause of syndromic disorders that include epilepsy as a feature, while microRNAs have had various roles ascribed to them, including as biomarkers and potential therapeutic targets.^19^ How non‐coding variation might interact with a variant of main effect in DEEs, or epilepsy more broadly, has not yet been systematically explored.

As a community, we have been very successful in identifying coding genes that carry variants in presumed monogenic epilepsies. However, it is likely that there is unaddressed complexity in many such types of epilepsy. It is important to explore this complexity with detailed evaluation of WGS, in combination with perspicacious phenotyping, to better understand disease biology, provide proper boundaries for resulting clinical phenotypes, and understand existing, and develop new, therapeutics.

### Genomic influences on treatment response and outcomes

Pharmacogenomics and precision medicine have received much attention in epilepsy over recent years. While WGS offers new opportunities in discovery, for example, through revealing disease biology and informing precision medicine approaches, the number of precision treatments proven to be effective in epilepsy remains very small. Nevertheless, this area of development from discovery of a monogenic cause to rational therapy is important, for example, with recent gene‐based approaches in clinical trials for several rare forms of epilepsy, including Dravet syndrome (such studies are available at ClinicalTrials.gov, https://clinicaltrials.gov). In many of these trials, a molecular genetic diagnosis for the condition, in addition to the clinical diagnosis, is an inclusion criterion. WGS can facilitate this and exclude co‐incidental rare variants that might compromise treatment. Sequencing might eventually identify variants that might predict a response beyond the variant, thus permitting trial inclusion. It is of course always worth keeping in mind that exclusion of variants in other genes is a dynamic exercise as new genes linked with epilepsy, and new methods of examining the genome, emerge that may pick out variants previously missed.

Currently, there are few pharmacogenomic variants known to be of clinical relevance in epilepsy. Such variants include those raising the risk of severe or mild cutaneous adverse reactions (e.g. HLA‐A*31:01, HLA‐B*15:02). These variants can be reliably identified in WGS data,^20^ meaning that anyone who has undergone WGS could have their genotypes at these loci determined, requiring only careful re‐examination of existing data. In a study of 1043 people with epilepsy who had undergone WGS through the UK 100,000 Genomes Project, four HLA‐B*15:02 and 86 HLA‐A*31:01 carriers were identified. Some of these individuals had already had serious cutaneous adverse reactions.^20^ For all individuals with epilepsy who have undergone WGS, retrospective inspection of existing data could uncover important pharmacogenomic variants, both those already known and others yet to be discovered.

For some people with epilepsy, focal resective neurosurgery may be a very effective treatment for seizure control. Historically, genomics have typically not been considered in presurgical multidisciplinary discussions,^21^ but now roles have been proposed for genomic information in presurgical evaluation. Rare germ line pathogenic or likely pathogenic genomic variants may influence outcomes,^22^ while testing brain tissue may reveal somatic causes.^23^, ^24^ Combining genetics with sophisticated histopathological analysis and transcriptomics can further deepen our understanding of epileptogenic lesions.^25^ Obtaining tissue for somatic testing can be difficult. Brain tissue is usually only available from patients who have epilepsy surgery or intracranial electroencephalogram (EEG) evaluation. Sometimes, other tissues may provide DNA for such testing, such as DNA from buccal swabs or from mosaic skin lesions.

Even if WGS does not reveal a monogenic cause for epilepsy in a surgical candidate, current analyses may still uncover variants that may influence surgical management broadly, such as variants in genes associated with risks of cardiac arrhythmia or upper‐airway structural dysmorphism.

In recognition of the value of genomic data in presurgical evaluation, some centres now routinely undertake WGS in all surgical candidates. Working the genome harder can mean that we consider WGS in individuals in whom it has not typically been considered (although usually for no good reason), looking for variants of clinical value beyond the discovery of a causal variant alone.

Overall, genetic testing may enhance more rounded management at the individual level, permitting more specific or precision treatment. New pathways for requesting tests, providing additional necessary information, analysing WGS data, and delivering a comprehensive report are needed.

### The value of putting genomics together with other data types

The genome, like a single pathogenic variant, is one element of a detailed characterization of an individual's epilepsy that it is now possible to determine. We can also examine the brain in detail for its structure (e.g. using magnetic resonance imaging [MRI]), its connectivity (e.g. using tractographic MRI), and function (e.g. using cognitive testing, functional MRI, EEG, magnetoencephalography, transcranial magnetic stimulation), among other investigational approaches. Putting these different elements together with genomics can bring out new layers of understanding of disease biology, of value to both research and immediate clinical perspectives. For example, PRS for epilepsy have been combined with imaging data, illuminating PRS‐related cortical thinning patterns anchored to distinct functional and structural network elements.^7^ Correlation of patterns of gene expression with robust measures of cortical thinning has identified microglial activation as a manipulable mechanism contributing to thinning.^26^ Other correlations have cast light on the potential basis for the spatial predilection of focal cortical dysplasia to the frontal lobes.^27^ Transcranial magnetic stimulation, a mesoscale tool allowing exploration of a level of phenotype between genetic variation and its clinical consequence, is being applied in preliminary studies to help understand, at the system level, why different variants in *SCN1A*, for example, can sometimes lead to Dravet syndrome and at other times to milder forms of epilepsy.

Combined studies of animal models and resected human brain tissue, using spatial multiomic techniques with genomics, have helped explore the functional consequences of somatic mutations in focal cortical dysplasia.^27^ Recently, somatic mutations have also begun to be explored in combination with highly resolved EEG data using the very small amounts of brain tissue adherent to explanted intracranial EEG electrodes used in clinical testing to better define the spatial localization of seizure foci.^28^ Currently, there are a few examples of the practical clinical implementation of combined multimodal data, perhaps best demonstrated by the inclusion of genetic data in multidisciplinary epilepsy surgery discussions and histopathological analyses. Eventually, we may have sufficient data and methods to begin to generate useful digital twins.

### Genome–environment interactions and addressing the dangers of climate change

The genome, like a single pathogenic variant within it, does not act in isolation. Multiple environmental factors (e.g. diet, exercise, gut microbiome, socioeconomic status) interacting with the genome can affect the final phenotypic manifestation. Another factor of growing importance, but still inadequately considered, is climate change. Through its many manifestations, such as increased frequency and severity of adverse weather events, climate change is likely to have multiple consequences for epilepsy.^29^ The effects of elevated, or rapidly changing, ambient temperatures, or fevers, on particular temperature‐sensitive forms of epilepsy such as Dravet syndrome, are well documented. In a proportion of a small number of adults undergoing intracranial EEG as part of an evaluation for possible surgical treatment for their treatment‐resistant epilepsy, both interictal discharge and seizure frequencies were elevated during heatwaves compared with non‐heatwave conditions. WGS showed that adults did not have a known genomic epilepsy, raising concerns that heatwaves may prove deleterious for a wider range of people with epilepsy, beyond those with known monogenic temperature‐sensitive types of epilepsy, and illustrating a new use of the genome.^30^ We undoubtedly need more information on the impacts of climate change on people with epilepsy, for example, by determining if common or rare genetic variation might render individuals more or less vulnerable to extreme heat. As climate change worsens, more uses of the genome are likely to emerge.

## CONCLUSION

**FIGURE 1:**
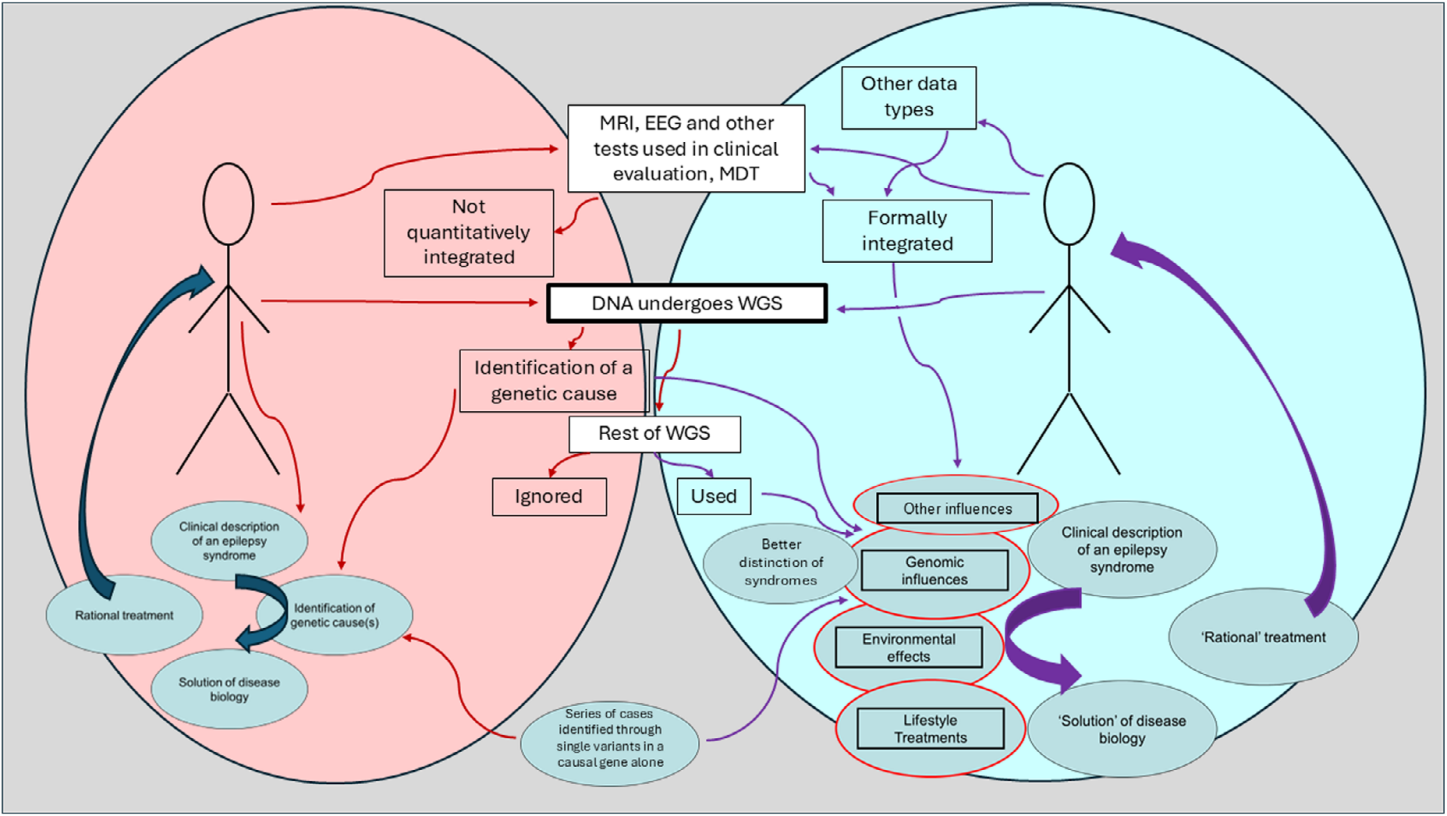
A current pattern of usage of genomic and other information is shown in the pink circle (left). Information from different modalities is typically not formally integrated but considered together in the expert review, for example, in a multidisciplinary team meeting. An alternative perspective considered in this review is shown in the blue circle (right). As much as possible of the remaining genome, not just the putatively causal variant, is used. It is formally integrated with other types of data to generate a better approximation of disease biology and rational treatment options; however, they are still shown within single quotation marks because even this approach is likely to fall short of a full understanding and treatment approach that tackles every difficulty faced by each individual. Abbreviations: EEG, electroencephalogram; MDT, multidisciplinary team; MRI, magnetic resonance imaging.

It is difficult to estimate how much information might be present in the human genome, and how much of that is useful for clinical and research purposes in epilepsy. Not only have many known features of the genome (e.g. somatic mosaicism, mobile elements, expanded repeats, modifying non‐coding variation, spatiotemporal patterns of expression and their environmental dependencies) not yet been systematically considered in most genomic studies, but there are probably questions we have not yet even thought to ask. Hopefully, the existence of a large and growing global WGS data set will allow rapid consideration of new questions as they arise. But even now, we can be clear that there is more information in the existing sequenced genome of people with epilepsy beyond a monogenic cause (if one is present). Given the resources and time, we can already work the existing sequenced genome harder (Figure 1). Next, we should consider whether everyone who receives a diagnosis of epilepsy should undergo WGS at diagnosis. The cost of sequencing is modest in comparison to that of other tests. The burden imposed on clinical genetics laboratories could be high if current reporting methods are used. Greater individual‐level expertise for clinicians, and greater provision of clinical scientist time, as well as new methods of reporting data for clinical implementation, are examples of improvements that are needed. Technical challenges also need to be addressed, for example, data harmonization (e.g. to account for different MRI scanners) and interoperability. We also need to think beyond our habitual silos. Experts in epilepsy imaging will need to consider genomics, while experts in genomics will need to think about EEG, and so on. Nevertheless, considering the range of information that we already know might be revealed, and its value, perhaps we could start thinking how this rich source of information could be accessed from the start of an individual's epilepsy journey to help map out that journey better and seek more informed, better outcomes from the outset.

## Data Availability

Data sharing not applicable ‐ no new data generated, or the article describes entirely theoretical research.
